# Supplementary material for: Mechanism of threonine ADP-ribosylation of F-actin by a Tc toxin
Source: Nat Commun. 2022 Jul 20;13:4202. doi: 10.1038/s41467-022-31836-w (PMC9300711; doi:10.1038/s41467-022-31836-w)
Supplement: Supplementary file 1 — Supplementary Information [file 41467_2022_31836_MOESM1_ESM.pdf]

Mechanism of threonine ADP-ribosylation of F-actin by a Tc toxin

Alexander Belyy, Florian Lindemann, Daniel Roderer, Johanna Funk, Benjamin Bardiaux, Jonas Protze, Peter Bieling, Hartmut Oschkinat and Stefan Raunser

Supplementary Information

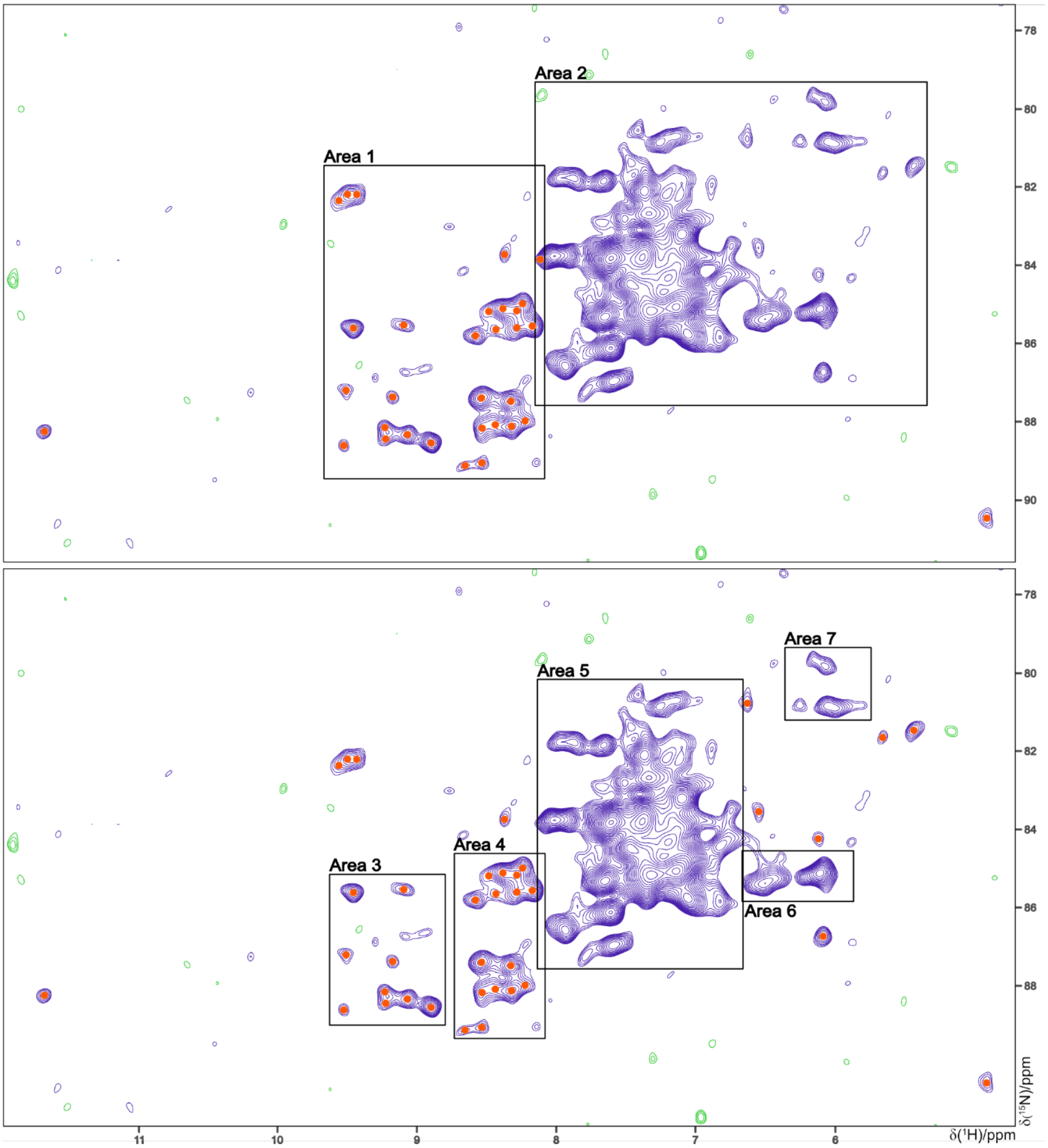

Supplementary Fig. 1. Arginine NεH cross peak region of a two-dimensional ^{1}H-^{15}N correlation spectrum of the TcB-TcC cocoon with two different signal integration schemes indicated by the frames. The different results obtained by counting the signals in Areas 1, 3, and 4 and taking the respective integrals as reference are listed in Supplementary Table 1. The red dots indicate the counted peaks. In all other cases only the integrals of the areas were used in the calculations. The peaks outside the frames in both panels are counted individually.

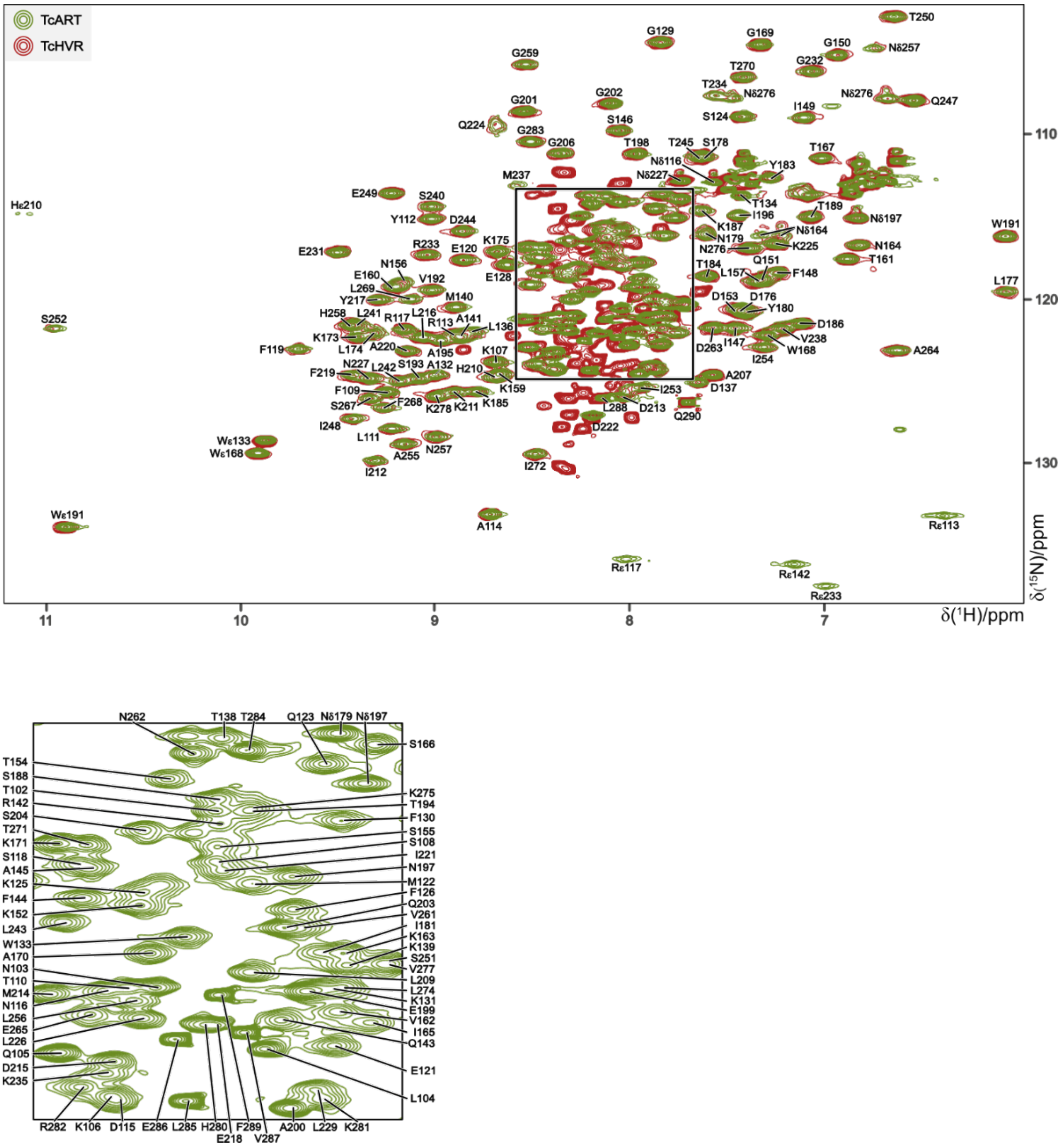

Supplementary Fig. 2. Overlay of two-dimensional ^{1}H-^{15}N correlation spectra of the TcHVR (red) and TcART (green) with assignments. The framed region in the TcART spectrum on top (δ(^{1}H): 7.7-8.6 ppm / δ(^{15}N): 113.2-124.8 ppm) is shown below in an enlarged manner. The non-overlapping red signals appear in the random coil ^{1}H chemical shift range between 7.7 and 8.5 ppm, indicating an unstructured N-terminus. In support, these signals are very large and show narrow line widths. Furthermore, no chemical shift changes of signals from residues in the globular domain are observed as it would occur if the N-terminus would interact.

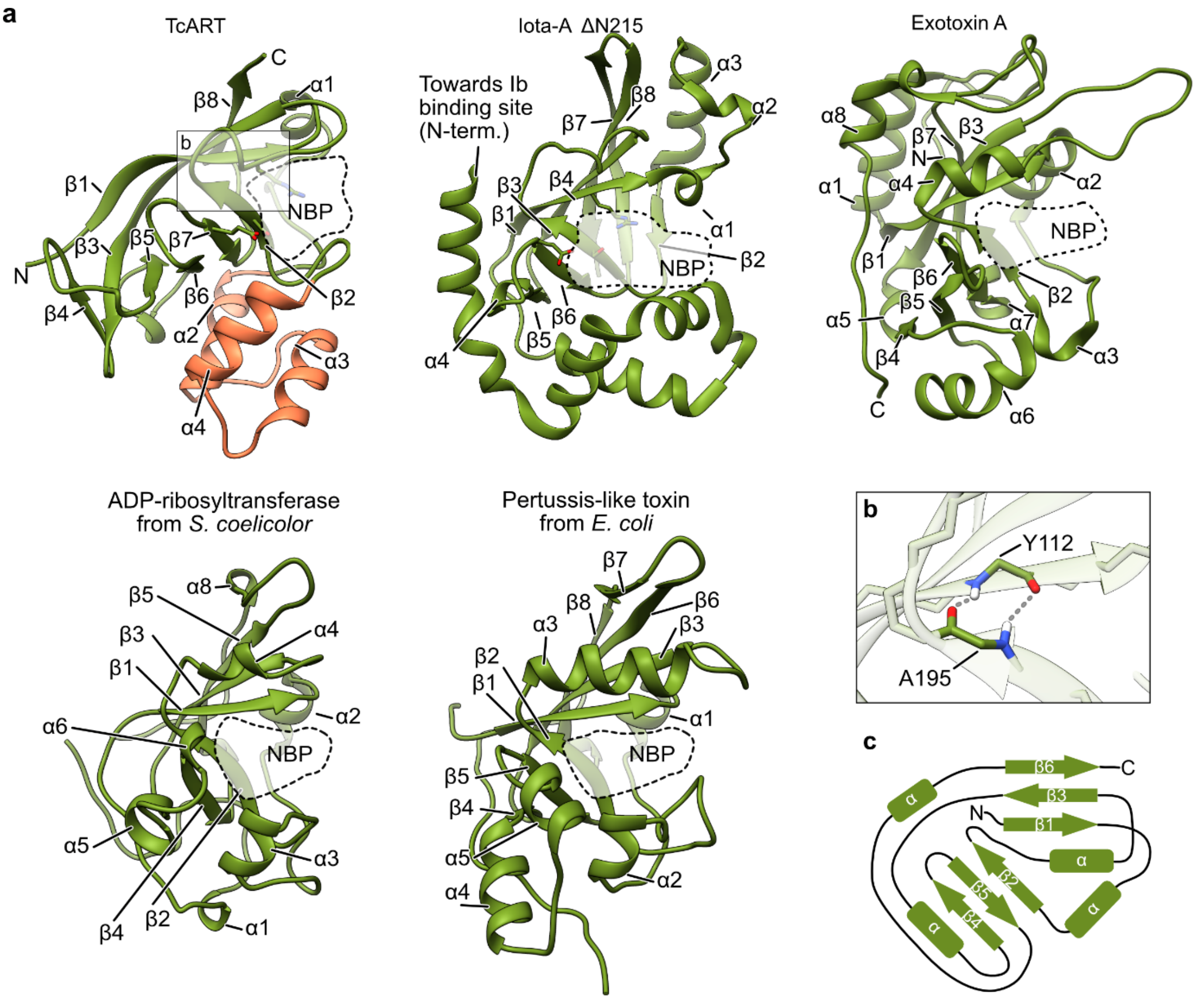

Supplementary Fig. 3. Comparison of TcART with homologous structures. a Comparison of atomic models of TcART**,** *C. perfringens* Iota toxin (PDB 1GIQ[1]), *P. aeruginosa* Exotoxin A (PDB 3B8H[2]), *S. coelicolor* ADP-ribosyltransferase (PDB 5ZJ5[3]) and *E. coli* pertussis-like toxin (PDB 4Z9D[4]). NBP: nucleotide binding pocket. In the TcART model, R113, S193 and E265 are shown. **b** Enlargement of the perpendicular connection between β1 and β2 in TcART where Y112 and A195 are involved in two hydrogen bonds. The same hydrogen bonds are present in the structural homologues shown. **c** Average secondary structure of the ART fold according to Aravind et al.[5].

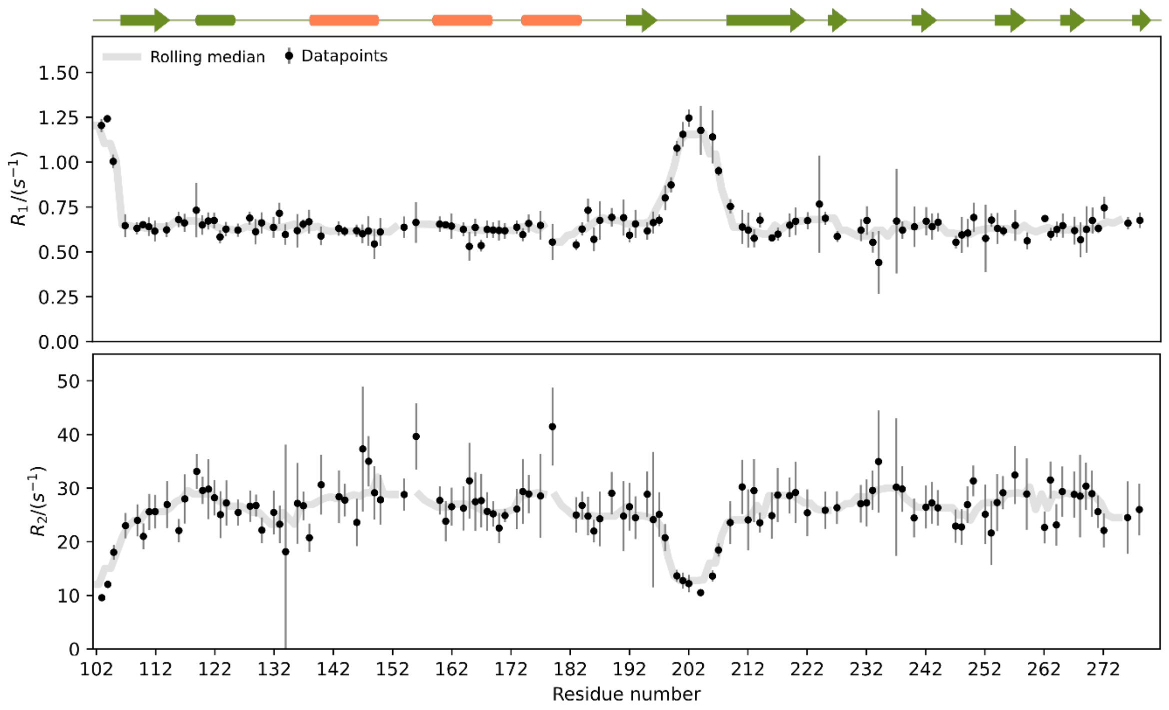

Supplementary Fig. 4. Longitudinal (top) and transverse (bottom) ^{15}N relaxation rates plotted versus the residue number. The secondary structure of TcART is given on top. Apart from the values of the N-terminal residues and those in the region 197-208 which indicate high flexibility the protein appears largely as a rigid, globular domain. The measurement was conducted at 600 MHz ^{1}H Larmor, a sample temperature of 280 K and with 500 µM protein in buffer (150 mM NaCl, 20 mM Hepes pH 7.8). Data points indicate the relaxation rate calculated by a least-squares fit of the experimental data to a mono exponential decay ($f(x) = a \cdot e^{-b \cdot x}$). Error bars indicate twice the standard deviation derived from the diagonal of the returned covariance matrix (pcov) and are a measure of the quality of the fit. Computation was performed using the curve_fit function of the python module SciPy. The rolling median over 7 data points is indicated in gray. Source Data are provided as a Source Data file and in BMRB entry 51478.

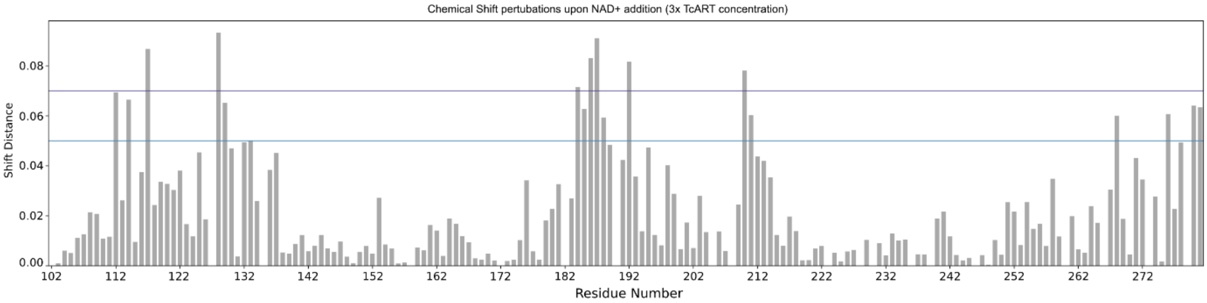

Supplementary Fig. 5. Chemical shift perturbations per residue within two-dimensional ^{1}H-^{15}N correlation spectra of TcART upon addition of a three-fold molar excess of NAD^{+}. The values above the blue and lilac lines are the basis for the surface coloring in Fig. 1d. Perturbations were quantified as a dimensionless number according to the formula given in the materials and methods part. Source data are provided as a Source Data file.

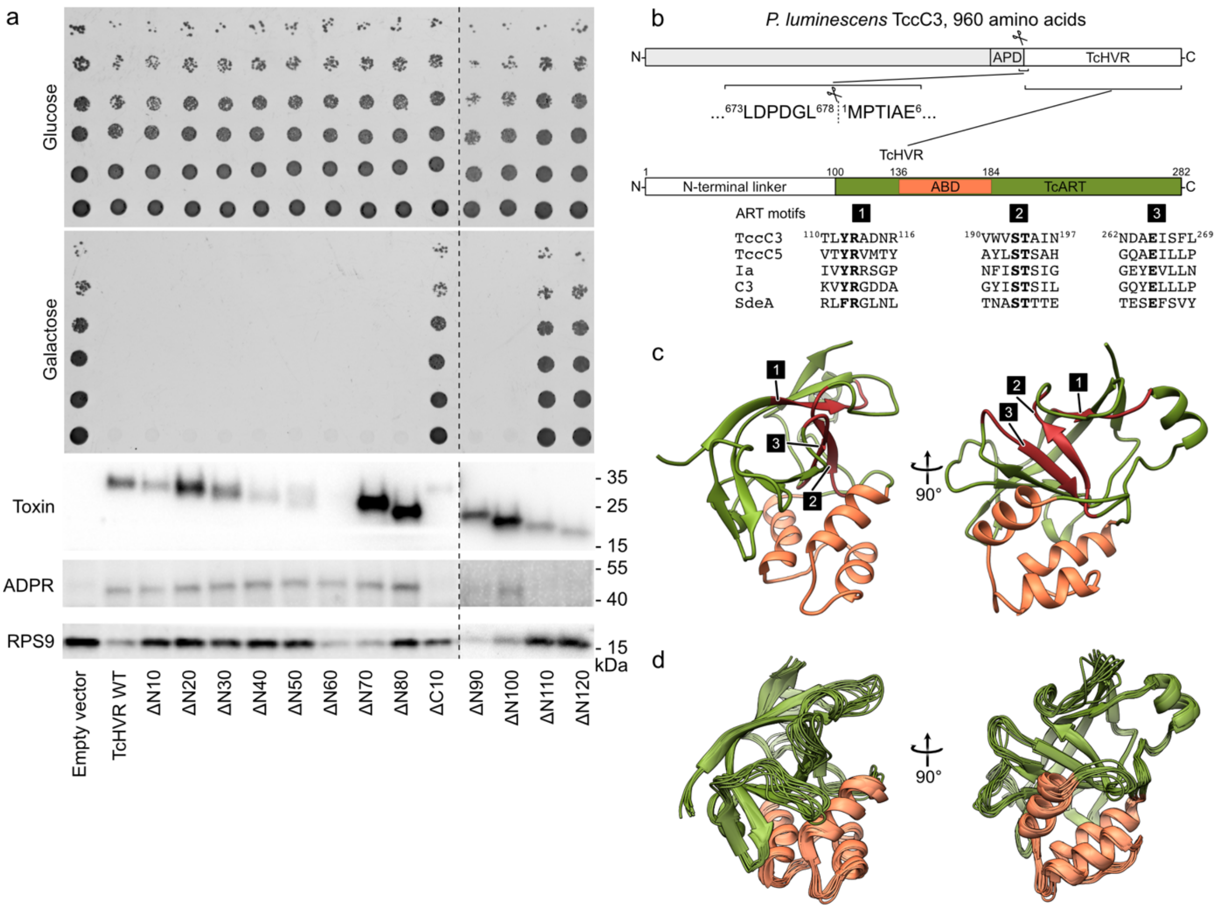

Supplementary Fig. 6. TcHVR is composed of the N-terminal linker and the C-terminal TcART. a Growth phenotype assay with *S. cerevisiae* expressing TcHVR variants under galactose promoter in the experimental conditions with low (Glucose) or high (Galactose) toxin expression. Analysis of protein expression and actin ADP-ribosylation was performed by western blot of cells grown on galactose-containing media with anti-myc (toxin), anti-ADP-ribose binding reagent (ADPR) and anti-ribosomal protein S9 (RPS9) antibodies. The panel is composed of two drop-test and western blot images as indicated by dashed lines. The experiment was performed once. **b** Schematic representation of the full-length TccC3, TcHVR and TcART. Sequences of 3 conserved ART motifs are provided for *P. luminescence* TccC3 and TccC5, *C. perfringens* Iota toxin, *Bacillus cereus* C3 toxin, *Legionella pneumophila* SdeA. APD – Aspartic protease domain. **c** Positions of ART motifs on the structure of TcART. **d** Superposition of the 10 lowest energy NMR structures.

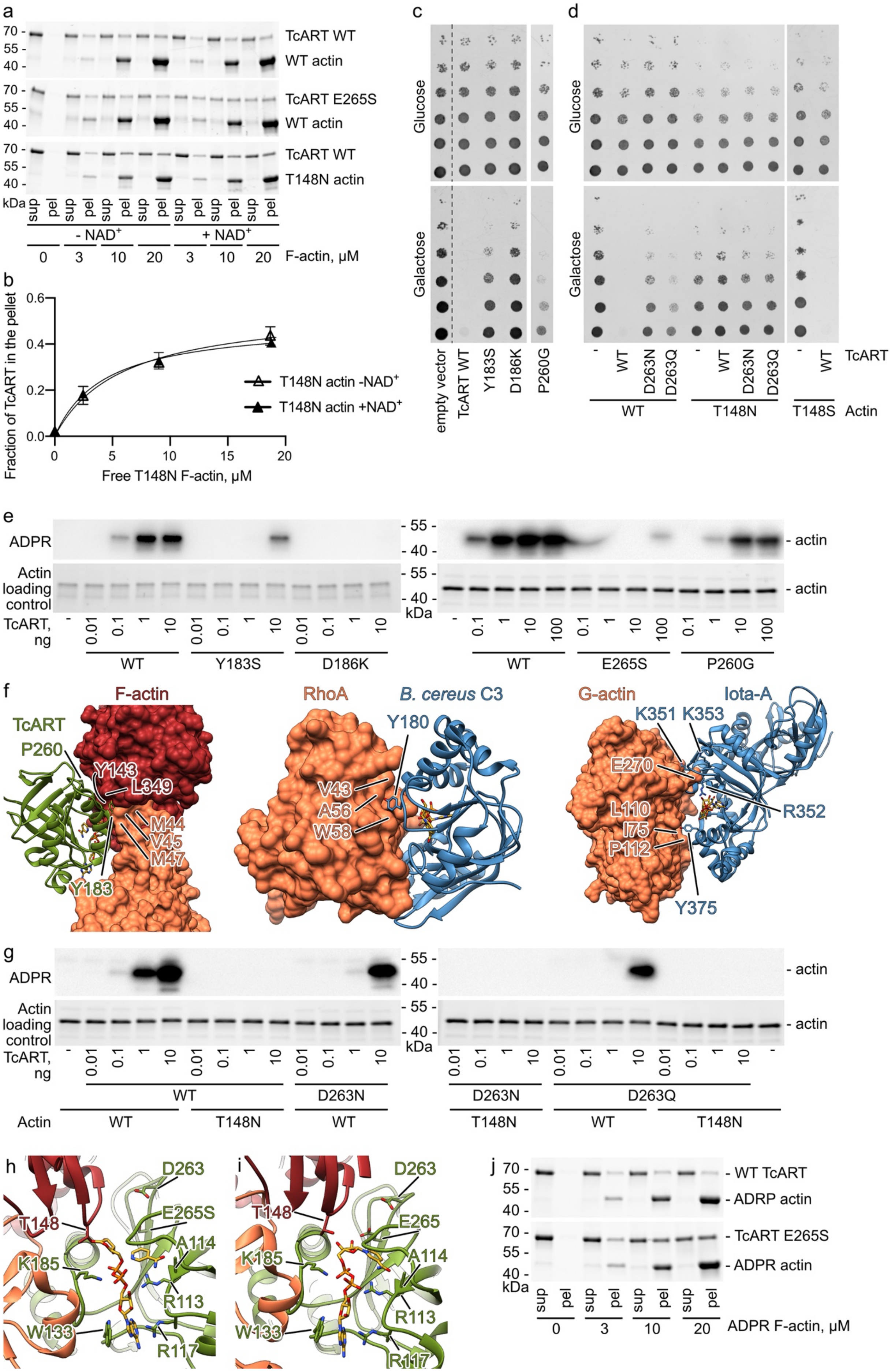

a
TcART WT
WT actin
TcART E265S
WT actin
TcART WT
T148N actin
kDa
sup
pel
- NAD+
+ NAD+
F-actin, µM
b
Fraction of TcART in the pellet
Free T148N F-actin, µM
T148N actin -NAD+
T148N actin +NAD+
c
Glucose
Galactose
empty vector
TcART WT
Y183S
D186K
P260G
d
TcART
Actin
WT
T148N
D263N
D263Q
T148S
e
ADPR
Actin loading control
TcART, ng
actin
WT
Y183S
D186K
E265S
P260G
f
F-actin
TcART
P260
Y143
L349
M44
V45
M47
Y183
RhoA
B. cereus C3
Y180
V43
A56
W58
G-actin
Iota-A
K351
K353
E270
R352
L110
I75
P112
Y375
g
ADPR
Actin loading control
TcART, ng
Actin
WT
T148N
D263N
D263Q
h
T148
D263
E265S
A114
K185
R113
W133
R117
i
T148
D263
E265
A114
K185
R113
W133
R117
j
WT TcART
ADRP actin
TcART E265S
ADPR actin
kDa
sup
pel
ADPR F-actin, µM

Supplementary Fig. 7. TcART-F-actin complex. a Co-sedimentation of phalloidin-stabilized F-actin (WT – rabbit muscle α actin, T148N – human cytosolic β actin) and 3 µM TcART analyzed by SDS-PAGE. The upper band corresponds to TcART variants, and the lower band corresponds to F-actin. Representative stain-free gels are shown. **b** The fractions of TcART that co-sedimented with F-actin were quantified by densitometry and plotted against F-actin concentrations. The data are presented as mean values, the error bars correspond to standard deviations of 3 independent experiments. **c** Growth phenotype assay with *S. cerevisiae* expressing WT actin and TcART variants under a strong galactose promoter in the experimental conditions with low (Glucose) or high (Galactose) toxin expression. **d** Growth phenotype assay with *S. cerevisiae* where WT actin was substituted to T148S and T148N variants expressing TcART variants under a strong galactose promoter in the experimental conditions with low (Glucose) or high (Galactose) toxin expression. **e**, **g** ADP-ribosylation (ADPR) of 1 µg of phalloidin-stabilized human cytosolic β-F-actin by TcART variants. The reaction mixture was first separated by SDS-PAGE (lower panel), blotted and developed with anti-ADPR reagent in western blot (upper panel). The experiments were performed twice. **f** Comparison of interfaces of the TcART-F-actin, *B. cereus* C3-RhoA (PDB 5BWM[6]) and *C. perfringens* Iota-A-G-actin (PDB 4H03[7]) complexes. **h** Cryo-EM model of the catalytic center of TcART in the post-reaction state. **i** Model of the catalytic center of TcART in the pre-reaction state. The model was obtained by docking of NAD^+ to the cryo-EM structure in the post-reaction state. **j** Co-sedimentation of pre-ADP-ribosylated phalloidin-stabilized rabbit muscle F-actin and 3 µM TcART analyzed by SDS-PAGE. The upper band corresponds to TcART variants, and the lower band corresponds to F-actin. Representative stain-free gels are shown. The experiment was performed three times. The average values are presented at Fig. 3e. Source data are provided as a Source Data file.

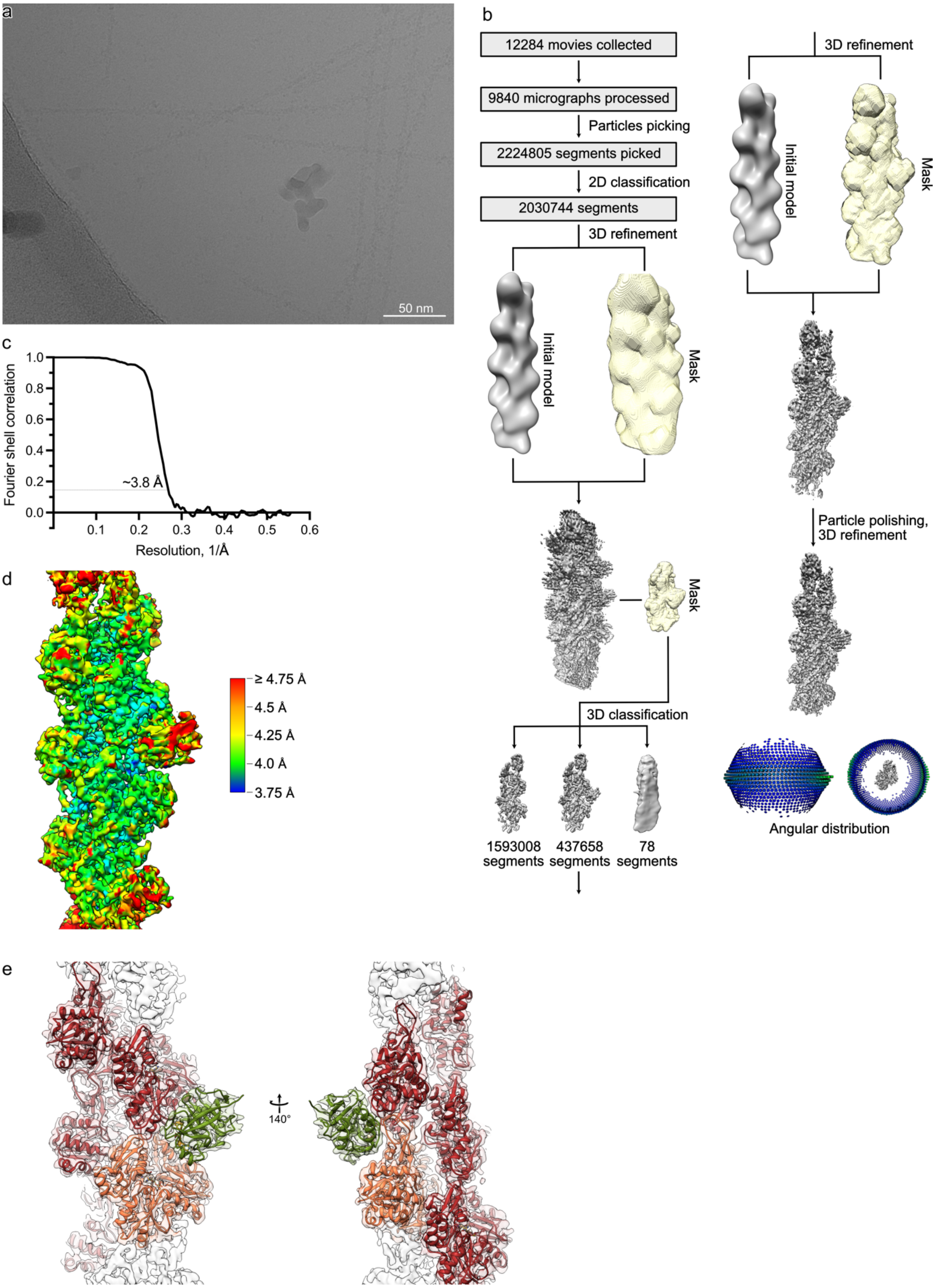

Supplementary Fig. 8. Processing of the TcART-F-actin complex. a An example of the 9840 analyzed cryo-EM micrographs. **b** Processing overview. **c** Fourier shell correlation curve of the final masked map calculated at 120-Å-long central section of the filament. **d** Local resolution gradient of the reconstruction. **e** Fit of the molecular model into the cryo-EM density. Source data are provided as a Source Data file.

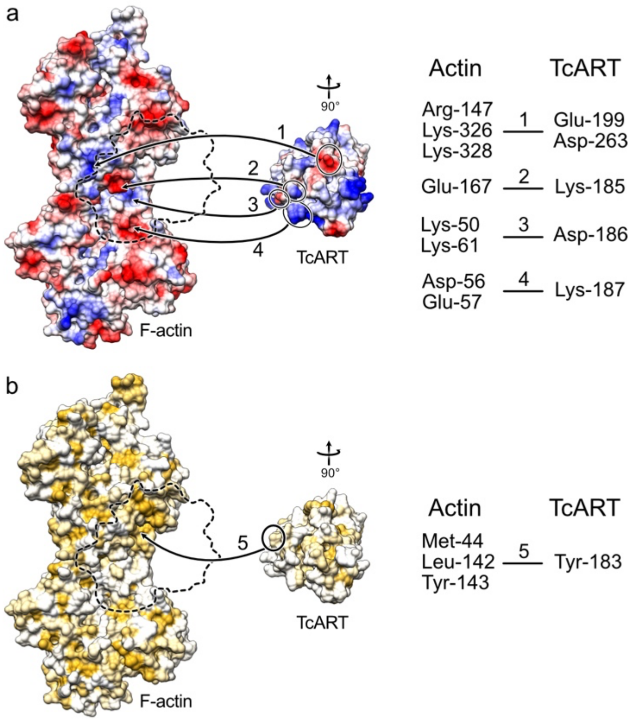

Supplementary Fig. 9. Interactions in the TcART-F-actin complex. a Electrostatic and **b** hydrophobic interactions in the TcART-F-actin complex.

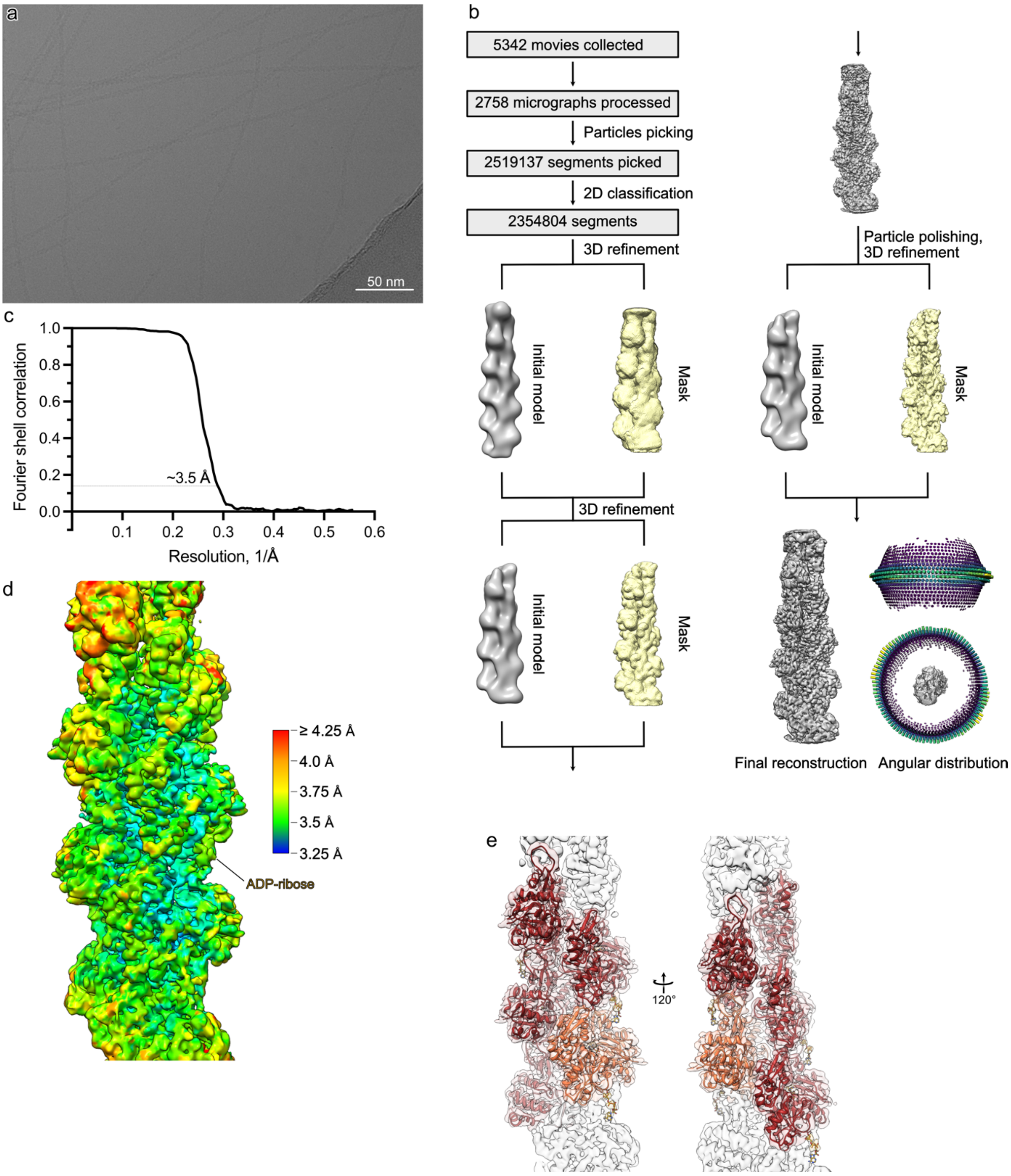

Supplementary Fig. 10. Processing of the ADP-ribosylated F-actin. a An example of the 2758 analyzed cryo-EM micrographs. **b** Processing overview. **c** Fourier shell correlation curve of the final masked map calculated at 120-Å-long central section of the filament. **d** Local resolution gradient of the reconstruction. **e** Fit of the molecular model into the cryo-EM density. Source data are provided as a Source Data file.

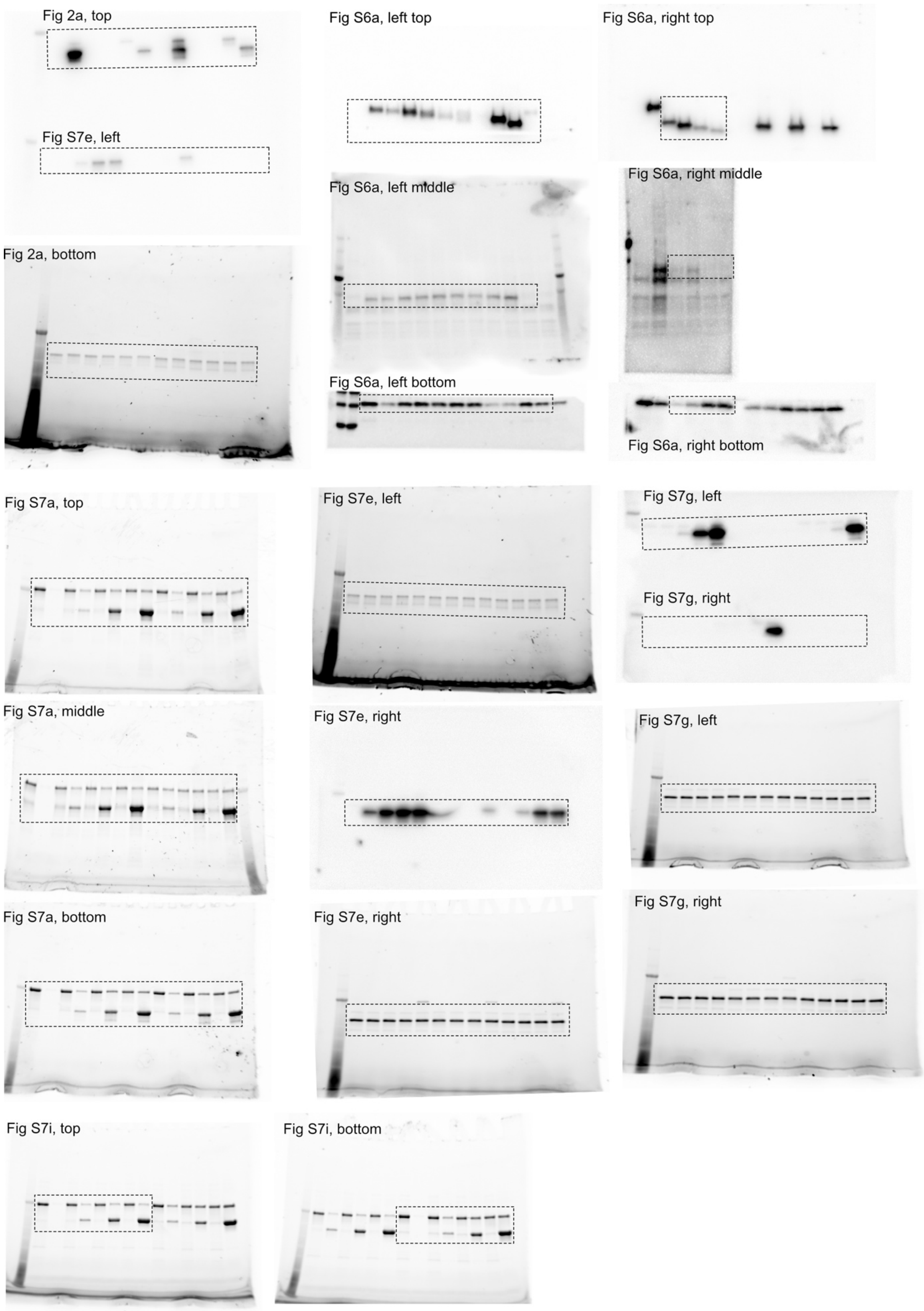

Supplementary Fig. 11. Uncropped gels and Western blots.

Supplementary Table 1. Integrals for indicated regions in Supplementary Fig. 1.

Ref. Area	Estimated Peaks	Area	Absolute Integral	Estimated Peaks		
1	30	1	5.50E+12	30.00		
		2	2.16E+13	117.55		
		-	exterior Peaks	2	Σ	149.55
		3	1.62E+12	8.84		
		4	2.87E+12	15.64		
		5	1.80E+13	98.13		
		6	1.19E+12	6.49		
		7	6.46E+11	3.52		
		-	exterior Peaks	12	Σ	144.62
3	9	1	5.50E+12	30.55		
		2	2.16E+13	119.69		
		-	exterior Peaks	2	Σ	152.24
		3	1.62E+12	9.00		
		4	2.87E+12	15.92		
		5	1.80E+13	99.92		
		6	1.19E+12	6.61		
		7	6.46E+11	3.59		
		-	exterior Peaks	12	Σ	147.04
4	16	1	5.50E+12	30.70		
		2	2.16E+13	120.27		
		-	exterior Peaks	2	Σ	152.97
		3	1.62E+12	9.04		
		4	2.87E+12	16.00		
		5	1.80E+13	100.41		
		6	1.19E+12	6.65		
		7	6.46E+11	3.60		
		-	exterior Peaks	12	Σ	147.70

Supplementary Table 2. Cryo-EM data collection, refinement, and validation statistics

Project	TcART-F-α-rabbit actin complex	ADPR-F-α-rabbit actin
Microscope	Titan Krios	
Voltage (kV)	300	
Defocus range (μm)	-0.5 to -2.5	
Camera	Gatan K3 (Superresolution mode)	
Pixel size (Å)	0.9	
Total electron dose (e/Å^2)	84.9	82.3
Exposure time (s)	4	
Frames per movie	60	
Number of movies	9840 (12284)	2758 (5342)
**3D Refinement**		
Number of particles	437658	2354804
Final resolution (Å)	3.8	3.5
Helical rise (Å)	28.1	28.2
Helical twist (°)	-167.1	-166.7
**Atomic model statistics**		
Non-hydrogen atoms	16102	14815
Molprobity score	2.00	1.55
Clashscore	16.81	3.75
EMRinger score	2.3	3.12
Bond RMSD (Å)	0.01	0.011
Angle RMSD (°)	1.29	1.18
Poor rotamers (%)	0.06	0
Favored rotamers (%)	96.47	97.44
Ramachandran favored (%)	96.47	94.26
Ramachandran allowed (%)	3.43	5.74
Ramachandran outliers (%)	0.1	0

Supplementary Table 3. List of primers, strains and plasmids used in this study.

**Bacterial and yeast strains**	**Description**	**Reference**
E. coli DH5α	F^- Φ80*lac*ZΔM15 Δ(*lac*ZYA-*arg*F) U169 *rec*A1 *end*A1 *hsd*R17(r_k^-, m_k^+) *pho*A *sup*E44 *thi*-1 *gyr*A96 *rel*A1 λ^-	Invitrogen
E. coli BL21 DE3 CodonPlus RIPL	F^- *ompT hsdS*(r_B^- m_B^-) *dcm*$^+$ Tet^r *gal*λ(DE3) *endA* Hte [*argU proL* Cam^r] [*argU ileY leuW* Strep/Spec^r]	Agilent
S. cerevisiae MH272-3fα	"Wild-type" strain*, ura3, leu2, his3, trp1, ade2*	[8]
S. cerevisiae SC483	*S. cerevisiae* MH272-3fα *act1::*LEU2 + *ACT1*[Ura3]	[9]
S. cerevisiae SC489	*S. cerevisiae* MH272-3fα *act1::*LEU2 + *ACT1*[His3]	[9]
S. cerevisiae Y395	*S. cerevisiae* MH272-3fα + empty vector [Ade] (2473)	[10]
S. cerevisiae Y547	*S. cerevisiae* MH272-3fα + WT TcHVR [Ade] (pB507)	This study
S. cerevisiae Y617	*S. cerevisiae* MH272-3fα + TcHVR ΔN10 [Ade] (pB596)	This study
S. cerevisiae Y618	*S. cerevisiae* MH272-3fα + TcHVR ΔN20 [Ade] (pB597)	This study
S. cerevisiae Y619	*S. cerevisiae* MH272-3fα + TcHVR ΔN30 [Ade] (pB598)	This study
S. cerevisiae Y620	*S. cerevisiae* MH272-3fα + TcHVR ΔN40 [Ade] (pB599)	This study
S. cerevisiae Y621	*S. cerevisiae* MH272-3fα + TcHVR ΔN50 [Ade] (pB600)	This study
S. cerevisiae Y622	*S. cerevisiae* MH272-3fα + TcHVR ΔN60 [Ade] (pB601)	This study
S. cerevisiae Y623	*S. cerevisiae* MH272-3fα + TcHVR ΔN70 [Ade] (pB602)	This study
S. cerevisiae Y624	*S. cerevisiae* MH272-3fα + TcHVR ΔN80 [Ade] (pB603)	This study
S. cerevisiae Y638	*S. cerevisiae* MH272-3fα + TcHVR ΔN90 [Ade] (pB630)	This study
S. cerevisiae Y639	*S. cerevisiae* MH272-3fα + TcHVR ΔN100 (ART) [Ade] (pB631)	This study
S. cerevisiae Y640	*S. cerevisiae* MH272-3fα + TcHVR ΔN110 [Ade] (pB632)	This study
S. cerevisiae Y641	*S. cerevisiae* MH272-3fα + TcHVR ΔN120 [Ade] (pB633)	This study
S. cerevisiae Y625	*S. cerevisiae* MH272-3fα + TcHVR ΔC10 [Ade] (pB604)	This study
S. cerevisiae Y626	*S. cerevisiae* MH272-3fα + TcHVR ΔC20 [Ade] (pB605)	This study
S. cerevisiae Y733	*S. cerevisiae* MH272-3fα + TcART Y183S [Ade] (pB751)	This study
S. cerevisiae Y735	*S. cerevisiae* MH272-3fα + TcART D186K [Ade] (pB753)	This study
S. cerevisiae Y738	*S. cerevisiae* MH272-3fα *act1::*LEU2 + *ACT1* T148S [His3] (pB748)	This study
S. cerevisiae Y739	*S. cerevisiae* MH272-3fα *act1::*LEU2 + *ACT1* T148N [His3] (pB749)	This study
S. cerevisiae Y740	*S. cerevisiae* MH272-3fα *act1::*LEU2 + *ACT1* [His3] + empty vector [Ade] (pB87)	This study
S. cerevisiae Y741	*S. cerevisiae* MH272-3fα *act1::*LEU2 + *ACT1* [His3] + TcART [Ade] (pB631)	This study
S. cerevisiae Y742	*S. cerevisiae* MH272-3fα *act1::*LEU2 + *ACT1* T148S[His3] + empty vector [Ade] (2473)	This study
S. cerevisiae Y743	*S. cerevisiae* MH272-3fα *act1::*LEU2 + *ACT1* T148S[His3] + TcART [Ade] (pB631)	This study
S. cerevisiae Y744	*S. cerevisiae* MH272-3fα *act1::*LEU2 + *ACT1* T148N [His3] + empty vector [Ade] (2473)	This study

S. cerevisiae Y745	*S. cerevisiae* MH272-3fα *act1::*LEU2 + *ACT1* T148N [His3] + TcART [Ade] (pB631)	This study
S. cerevisiae Y796	*S. cerevisiae* MH272-3fα *act1::*LEU2 + *ACT1*[His3] + TcART P260G [Ade] (pB849)	This study
S. cerevisiae Y797	*S. cerevisiae* MH272-3fα *act1::*LEU2 + *ACT1*[His3] + TcART D263N [Ade] (pB850)	This study
S. cerevisiae Y798	*S. cerevisiae* MH272-3fα *act1::*LEU2 + *ACT1*[His3] + TcART D263Q [Ade] (pB851)	This study
S. cerevisiae Y800	*S. cerevisiae* MH272-3fα *act1::*LEU2 + *ACT1* T148N [His3] + TcART D263N [Ade] (pB850)	This study
S. cerevisiae Y801	*S. cerevisiae* MH272-3fα *act1::*LEU2 + *ACT1* T148N [His3] + TcART D263Q [Ade] (pB851)	This study
**Plasmids for experiments in *S. cerevisiae***		
2473 YEpGal555	*E. coli*/*S. cerevisiae* shuttle vector [ADE2] with Gal1 promoter	[9]
pB507 TcHVR WT	TcHVR WT was amplified from p579 using oligonucleotides tatactcgagttaatgccaacaattgcagaacg and tatatagctagctctcttatgaggttttacatttttaagc. The PCR product was digested with XhoI and NheI and ligated into digested 2473 vector.	This study
pB596 TcHVR ΔN10	TcHVR with the N-terminal deletion was amplified from pB501 using oligonucleotides tatactcgagctaaaaaaaaataaagtaacagactcagcgcc and tatatagctagctctcttatgaggttttacatttttaagc. The PCR product was digested with XhoI and NheI and ligated into digested 2473 vector.	This study
pB597 TcHVR ΔN20	TcHVR with the N-terminal deletion was amplified from pB501 using oligonucleotides tatactcgagccttcgccagcaaatgcc and tatatagctagctctcttatgaggttttacatttttaagc. The PCR product was digested with XhoI and NheI and ligated into digested 2473 vector.	This study
pB598 TcHVR ΔN30	TcHVR with the N-terminal deletion was amplified from pB501 using oligonucleotides tatactcgagataaacatccgcccgcctgtag and tatatagctagctctcttatgaggttttacatttttaagc. The PCR product was digested with XhoI and NheI and ligated into digested 2473 vector.	This study
pB599 TcHVR ΔN40	TcHVR with the N-terminal deletion was amplified from pB501 using oligonucleotides tatactcgagcctagcttaccgaaagcatcaacg and tatatagctagctctcttatgaggttttacatttttaagc. The PCR product was digested with XhoI and NheI and ligated into digested 2473 vector.	This study
pB600 TcHVR ΔN50	TcHVR with the N-terminal deletion was amplified from pB501 using oligonucleotides tatactcgagcaaccaaccacacaccctatcg and tatatagctagctctcttatgaggttttacatttttaagc. The PCR product was digested with XhoI and NheI and ligated into digested 2473 vector.	This study
pB601 TcHVR ΔN60	TcHVR with the N-terminal deletion was amplified from pB501 using oligonucleotides tatactcgagaacataaaaccaacgacgtctggg and tatatagctagctctcttatgaggttttacatttttaagc. The PCR product was digested with XhoI and NheI and ligated into digested 2473 vector.	This study
pB602 TcHVR ΔN70	TcHVR with the N-terminal deletion was amplified from pB501 using oligonucleotides tatactcgagattgttgctccattgagtccagtag and	This study

	tatatagctagctctcttatgaggttttacatttttaagc. The PCR product was digested with XhoI and NheI and ligated into digested 2473 vector.	
pB603 TcHVR ΔN80	TcHVR with the N-terminal deletion was amplified from pB501 using oligonucleotides tatactcgagaaatctacttctgaaatctctctgccagaaag and tatatagctagctctcttatgaggttttacatttttaagc. The PCR product was digested with XhoI and NheI and ligated into digested 2473 vector.	This study
pB604 TcHVR ΔC10	TcHVR with the C-terminal deletion was amplified from pB501 using oligonucleotides tatactcgagttaatgccaacaattgcagaacg and tatatagctagcaattgttgtcagaaatgaaatttctgcatcatttaccgg. The PCR product was digested with XhoI and NheI and ligated into digested 2473 vector.	This study
pB605 TcHVR ΔC20	TcHVR with the C-terminal deletion was amplified from pB501 using oligonucleotides tatactcgagttaatgccaacaattgcagaacg and tatatagctagcatttaccggtccatgattaagtgcaatg. The PCR product was digested with XhoI and NheI and ligated into digested 2473 vector.	This study
pB630 TcHVR ΔN90	TcHVR with the N-terminal deletion was amplified from pB501 using oligonucleotides tatactcgagagcgctcaaagcagttcttcaagc and tatatagctagctctcttatgaggttttacatttttaagc. The PCR product was digested with XhoI and NheI and ligated into digested 2473 vector.	This study
pB631 TcHVR ΔN100 (TcART)	TcHVR with the N-terminal deletion was amplified from pB501 using oligonucleotides tatactcgagtcgacaaatctacagaaaaaatcatttac and tatatagctagctctcttatgaggttttacatttttaagc. The PCR product was digested with XhoI and NheI and ligated into digested 2473 vector.	This study
pB632 TcHVR ΔN110	TcHVR with the N-terminal deletion was amplified from pB501 using oligonucleotides tatactcgagttatatagagcagataacagatcc and tatatagctagctctcttatgaggttttacatttttaagc. The PCR product was digested with XhoI and NheI and ligated into digested 2473 vector.	This study
pB633 TcHVR ΔN120	TcHVR with the N-terminal deletion was amplified from pB501 using oligonucleotides tatactcgaggaaatgcaaagtaaattccctgaagg and tatatagctagctctcttatgaggttttacatttttaagc. The PCR product was digested with XhoI and NheI and ligated into digested 2473 vector.	This study
pB748 pRS313 Actin T148S	The T148S mutation was generated by two-step overlap PCR using oligonucleotides ctcttccggtagatctactggtattg, caggaaacagctatgacc, caataccagtagatctaccggaagag and ttcgtgataagtgatagtg. The PCR product was digested with ClaI and SalI and was used to exchange the WT ACT1 gene in 2475 p1387 pRS313 Actin [9].	This study
pB749 pRS313 Actin T148N	The T148N mutation was generated by two-step overlap PCR using oligonucleotides gtactcttccggtagaaacactggtattgttttgg, caggaaacagctatgacc, ccaaaacaataccagtgtttctaccggaagagtac and ttcgtgataagtgatagtg. The PCR product was digested with ClaI and SalI and was used to exchange the WT ACT1 gene in 2475 p1387 pRS313 Actin [9].	This study
pB751 TcART Y183S	The Y183S mutation was generated by two-step overlap PCR using oligonucleotides tatactcgagtcgacaaatctacagaaaaaatcatttac, tatatagctagctctcttatgaggttttacatttttaagc, caaattacataaaatctaccaaggac and	This study

	gtccttggtagattttatgtaatttg. The PCR product was digested with XhoI and NheI and ligated into digested 2473 vector.	
pB753 TcART D186K	The D186K mutation was generated by two-step overlap PCR using oligonucleotides tatactcgagtcgacaaatctacagaaaaaatcatttac, tatatagctagctctcttatgaggttttacatttttaagc, cataaaatataccaagaaaaaatctacagtatg and catactgtagattttttcttggtatattttatg. The PCR product was digested with XhoI and NheI and ligated into digested 2473 vector.	This study
pB849 TcART P260Q	TcART with the P260Q mutation was amplified from pB631 using oligonucleotides tatactcgagtcgacaaatctacagaaaaaatcatttac and atcttagctagctctcttatgaggttttacatttttaagcggaattgttgtcagaaatgaaatttctgcatcatttactcctccatgattaag. The PCR product was digested with XhoI and NheI and ligated into digested 2473 vector.	This study
pB850 TcART D263N	TcART with the D263N mutation was amplified from pB631 using oligonucleotides tatactcgagtcgacaaatctacagaaaaaatcatttac and atcttagctagctctcttatgaggttttacatttttaagcggaattgttgtcagaaatgaaatttctgcattatttaccgg. The PCR product was digested with XhoI and NheI and ligated into digested 2473 vector.	This study
pB851 TcART D263Q	TcART with the D263Q mutation was amplified from pB631 using oligonucleotides tatactcgagtcgacaaatctacagaaaaaatcatttac and atcttagctagctctcttatgaggttttacatttttaagcggaattgttgtcagaaatgaaatttctgcttgatttaccgg. The PCR product was digested with XhoI and NheI and ligated into digested 2473 vector.	This study
**Plasmids for protein expression in *E. coli***		
1330 pET28 Iota-A	*Clostridium perfringens* Iota-A with N-terminal His-tag in pET28 vector	[9]
579 pET19 TcHVR WT	TcHVR WT with C-terminal His-tag in pET19 vector	[11]
613 TcdB2-TccC3	Fusion construct of TcdB2–TccC3	[12]
pB137 pET28 MBP	Maltose-binding protein with N-terminal His-tag in pET28 vector	[10]
pB656 pET28 MBP-TcHVR ΔN100 (TcART)	TcHVR with the N-terminal deletion was amplified from p579 using oligonucleotides tatagagctctggttcgacaaatctacagaaaaaatcatttac and tataaagcttttatctcttatgaggttttacatttttaagc. The PCR product was digested with SacI and HindIII and ligated into digested pB137 vector.	This study
pB685 pET28 MBP-TcART E265S	The E265S mutation was generated by two-step overlap PCR using oligonucleotides tatagagctctggttcgacaaatctacagaaaaaatcatttac, tataaagcttttatctcttatgaggttttacatttttaagc, gtaaatgatgcatcaatttcatttc and gaaatgaaattgatgcatcatttac. The PCR product was digested with SacI and HindIII and ligated into digested pB137 vector.	This study
pB751 pET28 MBP-TcART Y183S	The Y183S mutation was generated by two-step overlap PCR using oligonucleotides tatagagctctggttcgacaaatctacagaaaaaatcatttac, tataaagcttttatctcttatgaggttttacatttttaagc, caaattacataaaatctaccaaggac and gtccttggtagattttatgtaatttg. The PCR product was digested with SacI and HindIII and ligated into digested pB137 vector.	This study
pB753 pET28 MBP-TcART D186K	The D186K mutation was generated by two-step overlap PCR using oligonucleotides tatagagctctggttcgacaaatctacagaaaaaatcatttac,	This study

	tataaagcttttatctcttatgaggttttacatttttaagc, cataaaatataccaagaaaaaatctacagtatg and catactgtagatttttttcttggtatattttatg. The PCR product was digested with SacI and HindIII and ligated into digested pB137 vector.	
pB846 pET28 MBP-TcART P260Q	TcART with the P260Q mutation was amplified from pB656 using oligonucleotides tatagagctctggttcgacaaatctacagaaaaaatcatttac and tataaagcttttatctcttatgaggttttacatttttaagcggaattgttgtcagaaatgaaatttctgcatcatttacacctccatgattaag. The PCR product was digested with SacI and HindIII and ligated into digested pB137 vector.	This study
pB847 pET28 MBP-TcART D263N	TcART with the D263N mutation was amplified from pB656 using oligonucleotides tatagagctctggttcgacaaatctacagaaaaaatcatttac and tataaagcttttatctcttatgaggttttacatttttaagcggaattgttgtcagaaatgaaatttctgcattatttaccggtc. The PCR product was digested with SacI and HindIII and ligated into digested pB137 vector.	This study
pB848 pET28 MBP-TcART D263Q	TcART with the D263Q mutation was amplified from pB656 using oligonucleotides tatagagctctggttcgacaaatctacagaaaaaatcatttac and tataaagcttttatctcttatgaggttttacatttttaagcggaattgttgtcagaaatgaaatttctgcttgatttaccggtc. The PCR product was digested with SacI and HindIII and ligated into digested pB137 vector.	This study
2013 pET19b cHis-3C TcHVR ΔN100 (TcART)	TcHVR with the N-terminal deletion was amplified from p579 and cloned into pET19b vector.	This study
**Plasmids for protein expression in insect cells**		
2336 pFL ACTB C272A	Human ActB with C272A mutation followed by the cleavable linker and thymosin β4	[13]
2723 pFL ACTB T148N C272A	T148N mutations was introduced by the QuikChange method using oligonucleotides ctgtacgcctctggccgtaacactggcatcgtgatggactcc and ggagtccatcacgatgccagtgttacggccagaggcgtacag and 2336 as a matrix	This study

Supplementary Table 4. Parameters of NMR data acquisition and processing.

	Experiment	MHz	(F3 x)F2 x F1*	scans	complex datapoints	d1 in s	expt time	aq time in ms	SW in ppm	mixing time	processing datapoints	processing window functions
3D	CCH TOCSY	600	H x C x C	16	1024 x 128 x 128	1	3d 8h	51.2 x 5.2 x 5.2	16.7 x 80.8 x 80.8	10 ms	2048 x 512 x 256	qsine 2.5 x qsine 2 x qsine 2
2D	CH D_2O	600	H x C	16	1024 x 256	1.3	1h 35min	51.2 x 10.5	16.7 x 80.8	-	4096 x 2048	qsine 2 x qsine 2
3D	CHH NOESY aliphatic	750	H x C x H	8	1024 x 164 x 300	1.3	6d 14h	41 x 5.6 x 15.0	16.7 x 78 x 13.3	80 ms	1024 x 512 x 512	qsine 2 x qsine 2 x qsine 2
3D	CHH NOESY aliphatic NAD+	600	H x C x H	16	1024 x 128 x 73	1.3	2d 12h	34.1 x 5.5 x 12.1	25 x 77 x 15.1	80 ms	2048 x 512 x 512	qsine 2 x qsine 2 x qsine 2
3D	CHH NOESY aliphatic D_2O	600	H x C x H	8	1024 x 128 x 256	1.3	3d 18h	51.2 x 5.2 x 15.4	16.7 x 80.8 x 13.9	40 ms	2048 x 512 x 256	qsine 3 x qsine 2 x qsine 2
3D	CHH NOESY aromatic	750	H x C x H	8	1024 x 150 x 320	1.3	6d 10h	41 x 5.1 x 16	16.7 x 78 x 13.3	80 ms	2048 x 512 x 1024	qsine 3 x qsine 2 x qsine 3
2D	HH NOESY D_2O Ndec	600	H x H	80	2048 x 1024	1.3	1d 10h	102.4 x 6.1	16.7 x 13.9	40 ms	4096 x 4096	gm (-40.0 | 0.1) x qsine 3
2D	HN D_2O	600	H x N	32	1024 x 256	0.1	25 min	51.2 x 42.5	16.7 x 49.5	-	4096 x 1024	qsine 3 x qsine 2
2D	HN prot	600	H x N	4	2048 x 512	1.3	50 min	102.4 x 6.1	16.7 x 49.5	-	4096 x 1024	qsine 3 x qsine 3
2D	HN trosy	600	H x N	32	1024 x 1024	1.3	13h 16min	51.2 x 17	16.7 x 49.5	-	4096 x 2048	qsine 2.5 x qsine 2
3D	HNCA prot	600	H x N x C	32	1024 x 128 x 100	1.3	3d 10h	51.2 x 21.2 x 7.1	16.7 x 49.5 x 46.7	-	4096 x 256 x 256	qsine 3 x qsine 2 x qsine 2
3D	HNCACB	600	H x N x C	16	1024 x 80 x 140	1.3	3d 7h	51.2 x 16 x 7	16.7 x 41.1 x 66.3	-	1024 x 512 x 512	qsine 2.5 x qsine 2 x qsine 2
3D	HNCO	600	H x N x C	8	1024 x 80 x 128	1.3	1d 9h	51.2 x 16 x 25.6	16.7 x 41.1 x 16.6	-	1024 x 512 x 512	qsine 2.5 x qsine 2 x qsine 2
3D	HNCOCACB	600	H x N x C	16	1024 x 80 x 140	1.3	3d 8h	51.2 x 16 x 7	16.7 x 41.1 x 66.3	-	1024 x 512 x 512	qsine 2.5 x qsine 2 x qsine 2
3D	NHH NOESY 80ms mixing	750	H x N x H	8	1024 x 128 x 300	1.3	5d 4h	41 x 20.5 x 15	16.7 x 41.1 x 13.3	80 ms	2048 x 256 x 512	qsine 3 x qsine 2 x qsine 3
2D	NH + 0 mM NAD+ (= HN prot)	600	H x N	4	2048 x 512	1.3	50 min	102.4 x 6.1	16.7 x 49.5	-	4096 x 2048	qsine 3 x qsine 3
2D	NH + 1 mM NAD+	600	H x N	4	1024 x 256	1.3	24 min	51.2 x 42.5	16.7 x 49.5	-	4096 x 2048	qsine 3 x qsine 3
pseudo 3D	T1 relaxation	600	H x N x t1	32	1024 x 256 x 8	1.3	2d 5h	51.2 x 42.5 x 1500	16.7 x 49.5 x 0	-	4096 x 1024	qsine 2 x qsine 2
pseudo 3D	T2 relaxation	600	H x N x t1	32	1024 x 256 x 8	1.3	1d 6h	51.2 x 42.5 x 220	16.7 x 49.5 x 0	-	4096 x 1024	qsine 2 x qsine 2
2D	NH of cocoon without toxin inside	900	H x N	32	2048 x 1200	0.98		28.7 x 48.0	39.7 x 137	-	4096 x 4096	gm (-20 | 0.1) x qsine 3
											4096 x 4096	gm (-30 | 0.04) x qsine 2
2D	NH of cocoon with toxin inside	900	H x N	32	2048 x 1200	1		28.7 x 48.0	39.7 x 137	-	4096 x 4096	gm (-20 | 0.1) x qsine 3
											4096 x 4096	gm (-30 | 0.04) x qsine 2

*highest dimension is always the direct one

Supplementary Table 5. Summary of structural statistics for the NMR ensemble.

Completeness of resonance assignment			
	Backbone[a]		91%
	Sidechain non-H		74%
	Sidechain H		89%
	Aromatic		89%
Conformationally restricting restraints (consensus ensembles)			
	Distance restraints		
		Total	3315
		Intraresidue (i=j)	0
		Sequential ($\vert i-1 \vert=1$)	834
		Medium range ($1<\vert i-j \vert<5$)	460
		Long range ($\vert i-j \vert>=5$)	1297
		Ambiguous	724
		Dihedral angle restr.	306
		Hydrogen-bond restr.	37
Residual restraint violations (average of 2 consensus runs)			
	Average no. of distance viol. per structure		
		0.1 - 0.3 Å	209
		0.3 - 0.5 Å	165
		> 0.5 Å	270
	Average no. of dihedral viol. per structure		
		> 5°	3.6
Model quality[b]			
	RMSD backbone atoms (Å)[c]		0.5
	RMSD heavy atoms (Å)[c]		0.8
	RMSD bond lenghts (Å)		0.005
	RMSD bond angles (°)		0.7
Molprobity Ramachandran Statistics[b,c]			
	Most favored regions (%)		97.2
	Allowed regions (%)		2.7
	Disallowed regions (%)		0.1
Global quality scores (raw/Z scores)[b]			
	Verify3D		(0.25/-3.37)
	Prosall		(0.60/-0.21)
	Procheck (ϕ-ψ)[c]		(-0.42/-1.34)
	Procheck (all)[c]		(-0.35/-2.07)
	MolProbity clash score		(24.08/-2.61)
Model contents			
	Ordered residue ranges[b]		103-200, 207-231, 237-258, 260-280
	Total no. of residues		180
	BMRB accession number		34717, 51438
	PDB ID Code		7ZBQ

[a]CO resonances were only assigned for the deuterated protein

[b]calculated using the PSVS webserver (https://montelionelab.chem.rpi.edu/PSVS/PSVS/)

[c]for all ordered residues from PSVS based on dihedral order parameter $S(\phi)+S(\psi)>=1.8$

Supplementary References

1. Tsuge, H. *et al.* Crystal structure and site-directed mutagenesis of enzymatic components from Clostridium perfringens iota-toxin. *J Mol Biol* **325,** 471–483 (2003).
2. Jørgensen, R., Wang, Y., Visschedyk, D. & Merrill, A. R. The nature and character of the transition state for the ADP-ribosyltransferase reaction. *EMBO Rep* **9,** 802–809 (2008).
3. Yoshida, T. & Tsuge, H. Substrate N(2) atom recognition mechanism in pierisin family DNA-targeting, guanine-specific ADP-ribosyltransferase ScARP. *J Biol Chem* **293,** 13768–13774 (2018).
4. Littler, D. R. *et al.* Structure-function analyses of a pertussis-like toxin from pathogenic Escherichia coli reveal a distinct mechanism of inhibition of trimeric G-proteins. *J Biol Chem* **292,** 15143–15158 (2017).
5. Aravind, L., Zhang, D., de Souza, R. F., Anand, S. & Iyer, L. M. The natural history of ADP-ribosyltransferases and the ADP-ribosylation system. *Curr Top Microbiol Immunol* **384,** 3–32 (2015).
6. Toda, A., Tsurumura, T., Yoshida, T., Tsumori, Y. & Tsuge, H. Rho GTPase Recognition by C3 Exoenzyme Based on C3-RhoA Complex Structure. *J Biol Chem* **290,** 19423–19432 (2015).
7. Tsurumura, T. *et al.* Arginine ADP-ribosylation mechanism based on structural snapshots of iota-toxin and actin complex. *Proc Natl Acad Sci U S A* **110,** 4267–4272 (2013).
8. Heitman, J., Movva, N. R., Hiestand, P. C. & Hall, M. N. FK 506-binding protein proline rotamase is a target for the immunosuppressive agent FK 506 in Saccharomyces cerevisiae. *Proc Natl Acad Sci USA* **88,** 1948 (1991).
9. Belyy, A. *et al.* Roles of Asp179 and Glu270 in ADP-Ribosylation of Actin by Clostridium perfringens Iota Toxin. *PLoS One* **10,** e0145708 (2015).
10. Belyy, A. *et al.* The extreme C terminus of the Pseudomonas aeruginosa effector ExoY is crucial for binding to its eukaryotic activator, F-actin. *J Biol Chem* **293,** 19785–19796 (2018).
11. Roderer, D., Schubert, E., Sitsel, O. & Raunser, S. Towards the application of Tc toxins as a universal protein translocation system. *Nat Commun* **10,** 5263 (2019).
12. Gatsogiannis, C. *et al.* Tc toxin activation requires unfolding and refolding of a β-propeller. *Nature* **563,** 209–213 (2018).
13. Belyy, A., Merino, F., Mechold, U. & Raunser, S. Mechanism of actin-dependent activation of nucleotidyl cyclase toxins from bacterial human pathogens. *Nat Commun* **12,** 6628 (2021).
