# Supplementary material for: Mechanism of threonine ADP-ribosylation of F-actin by a Tc toxin
Source: Nat Commun. 2022 Jul 20;13:4202. doi: 10.1038/s41467-022-31836-w (PMC9300711; doi:10.1038/s41467-022-31836-w)
Supplement: Supplementary file 3 — Description of Additional Supplementary Files [file 41467_2022_31836_MOESM3_ESM.docx]

**Mechanism of threonine ADP-ribosylation of F-actin by a Tc toxin**

Alexander Belyy, Florian Lindemann, Daniel Roderer, Johanna Funk, Benjamin Bardiaux, Jonas Protze, Peter Bieling, Hartmut Oschkinat and Stefan Raunser

Description of Additional Supplementary Files

File Name: Supplementary Movie 1

Description: Mechanism of threonine ADP-ribosylation of F-actin by a Tc toxin.
