# Supplementary material for: Mechanism of threonine ADP-ribosylation of F-actin by a Tc toxin
Source: Nat Commun. 2022 Jul 20;13:4202. doi: 10.1038/s41467-022-31836-w (PMC9300711; doi:10.1038/s41467-022-31836-w)
Supplement: Supplementary file 5 — Reporting Summary [file 41467_2022_31836_MOESM5_ESM.pdf]

nature portfolio

Corresponding author(s): Hartmut Oschkinat, Stefan Raunser

Last updated by author(s): Jun 25, 2022

# Reporting Summary

Nature Portfolio wishes to improve the reproducibility of the work that we publish. This form provides structure for consistency and transparency in reporting. For further information on Nature Portfolio policies, see our Editorial Policies and the Editorial Policy Checklist.

## Statistics

For all statistical analyses, confirm that the following items are present in the figure legend, table legend, main text, or Methods section.

| n/a | Confirmed | |
|---|---|---|
| ☐ | ☒ | The exact sample size (*n*) for each experimental group/condition, given as a discrete number and unit of measurement |
| ☐ | ☒ | A statement on whether measurements were taken from distinct samples or whether the same sample was measured repeatedly |
| ☒ | ☐ | The statistical test(s) used AND whether they are one- or two-sided<br>*Only common tests should be described solely by name; describe more complex techniques in the Methods section.* |
| ☒ | ☐ | A description of all covariates tested |
| ☒ | ☐ | A description of any assumptions or corrections, such as tests of normality and adjustment for multiple comparisons |
| ☐ | ☒ | A full description of the statistical parameters including central tendency (e.g. means) or other basic estimates (e.g. regression coefficient) AND variation (e.g. standard deviation) or associated estimates of uncertainty (e.g. confidence intervals) |
| ☒ | ☐ | For null hypothesis testing, the test statistic (e.g. *F*, *t*, *r*) with confidence intervals, effect sizes, degrees of freedom and *P* value noted<br>*Give P values as exact values whenever suitable.* |
| ☒ | ☐ | For Bayesian analysis, information on the choice of priors and Markov chain Monte Carlo settings |
| ☒ | ☐ | For hierarchical and complex designs, identification of the appropriate level for tests and full reporting of outcomes |
| ☒ | ☐ | Estimates of effect sizes (e.g. Cohen's *d*, Pearson's *r*), indicating how they were calculated |

*Our web collection on statistics for biologists contains articles on many of the points above.*

## Software and code

Policy information about availability of computer code

| | |
|---|---|
| Data collection | - Cryo-EM data: EPU version 2.7 and 2.8 (Thermo Fisher Scientific)<br>- SDS-PAGE and western blots: ImageLab version 5.2.1<br>- NMR: TopSpin 3.5pl6, TopSpin 4.1.0<br>- TIRF microscopy: Nikon Elements AR 4.50 |
| Data analysis | - Cryo-EM data: crYOLO version 1.8; CTFFIND version 4.1.13; MotionCor2 version 1.3; TranSPHIRE versions 1.4.28, 1.5.13; SPHIRE versions 1.3, 1.4; Relion versions 3, 3.1; PHENIX version 1.17; ISOLDE version 1.0B4; UCSF Chimera version 1.14.<br>- Analysis of cosedimentation assays: Prism version 9, ImageLab version 5.2.1<br>- NMR: TopSpin 3.5pl6, TopSpin 4.1.0, CCPN Analysis 2.4.2 (assignment+peak picking), aria 2.3.2 and cns 1.21 (structure calculation)<br>- Docking: Glide included in the Maestro 12v7 software package (Schrödinger, LLC)<br>- Analysis of TIRFM images: Fiji 1.53C |

For manuscripts utilizing custom algorithms or software that are central to the research but not yet described in published literature, software must be made available to editors and reviewers. We strongly encourage code deposition in a community repository (e.g. GitHub). See the Nature Portfolio guidelines for submitting code & software for further information.

## Data

Policy information about availability of data

All manuscripts must include a data availability statement. This statement should provide the following information, where applicable:

- Accession codes, unique identifiers, or web links for publicly available datasets
- A description of any restrictions on data availability
- For clinical datasets or third party data, please ensure that the statement adheres to our policy

The coordinates for the cryo-EM structures of the TcART-F-actin complex and ADPR-F-actin have been deposited in the Electron Microscopy Data Bank under accession numbers EMD-14532 [https://www.ebi.ac.uk/pdbe/entry/emdb/EMD-14532] and 14533 [https://www.ebi.ac.uk/pdbe/entry/emdb/EMD-14533]. The corresponding molecular models for TcHVR, ADPR-F-actin, and the TcART-F-actin complex have been deposited at the wwPDB with accession codes PDB 7ZBQ [http://doi.org/10.2210/pdb7ZBQ/pdb], 7Z7H [http://doi.org/10.2210/pdb7Z7H/pdb] and 7Z7I [http://doi.org/10.2210/pdb7Z7I/pdb]. The NMR datasets used in this study are available in the BMRB under accession codes 34717 [https://bmrb.io/data_library/summary/index.php?bmrbId=34717] (Assignment data of protonated TcART), 51438 [https://bmrb.io/data_library/summary/index.php?bmrbId=51438] (Assignment data of deuterated TcART) and 51478 [https://bmrb.io/data_library/summary/index.php?bmrbId=51478] (Relaxation data of TcART). The raw data generated during the current study are available from the corresponding authors on reasonable request. Source data are provided with this paper. Uncropped gels and Western blots from Fig. 2a, S6 and S7 can be found in Supplementary Fig. 11. We used the following previously published structures: 1GIQ [http://doi.org/10.2210/pdb1GIQ/pdb], 5ZJ5 [http://doi.org/10.2210/pdb5ZJ5/pdb], 4Z9D [http://doi.org/10.2210/pdb4Z9D/pdb], 4TLV [http://doi.org/10.2210/pdb4TLV/pdb], 1PTO [http://doi.org/10.2210/pdb1PTO/pdb], 1WFX [http://doi.org/10.2210/pdb1WFX/pdb], 6E3A [http://doi.org/10.2210/pdb6E3A/pdb], 6RO0 [http://doi.org/10.2210/pdb6RO0/pdb], 5ONV [http://doi.org/10.2210/pdb5ONV/pdb], 3B8H [http://doi.org/10.2210/pdb3B8H/pdb], 5BWM [http://doi.org/10.2210/pdb5BWM/pdb], 4H03 [http://doi.org/10.2210/pdb4H03/pdb].

# Field-specific reporting

Please select the one below that is the best fit for your research. If you are not sure, read the appropriate sections before making your selection.

- [x] Life sciences
- [ ] Behavioural & social sciences
- [ ] Ecological, evolutionary & environmental sciences

For a reference copy of the document with all sections, see nature.com/documents/nr-reporting-summary-flat.pdf

# Life sciences study design

All studies must disclose on these points even when the disclosure is negative.

| | |
|---|---|
| Sample size | n=3; Sample size was chosen based on the previous publications (Belyy et al., JBC 2018, Belyy et al., Nat Commun 2016, 2021) |
| Data exclusions | No data were excluded. |
| Replication | Cosedimentation assays and TIRF microscopy assays were performed independently three times. ADP-ribosylation assays - twice. Analysis of protein expression and level of actin ADP-ribosylation was performed once.<br>All attempts at replication were successful. |
| Randomization | No randomization was necessary as all data, which passed quiality control, were used for analysis. Covariates were not controlled. |
| Blinding | The study does not contain experiments where blinding would be applicable. |

# Reporting for specific materials, systems and methods

We require information from authors about some types of materials, experimental systems and methods used in many studies. Here, indicate whether each material, system or method listed is relevant to your study. If you are not sure if a list item applies to your research, read the appropriate section before selecting a response.

## Materials & experimental systems

| n/a | Involved in the study | |
|---|---|---|
| [ ] | [x] | Antibodies |
| [ ] | [x] | Eukaryotic cell lines |
| [x] | [ ] | Palaeontology and archaeology |
| [x] | [ ] | Animals and other organisms |
| [x] | [ ] | Human research participants |
| [x] | [ ] | Clinical data |
| [x] | [ ] | Dual use research of concern |

## Methods

| n/a | Involved in the study | |
|---|---|---|
| [x] | [ ] | ChIP-seq |
| [x] | [ ] | Flow cytometry |
| [x] | [ ] | MRI-based neuroimaging |

## Antibodies

| | |
|---|---|
| Antibodies used | myc-tag (9B11, reference #2276) mouse mAb Cell signaling technology, lot 24, reference 02/2019, dilution 1:10000; RPS9 polyclonal rabbit antibody (used at dilution 1:10000), produced by Eurogentec, is a present of Prof. S. Rospert (University of Freiburg); Anti-pan-ADP-ribose binding reagent (reference #MABE1016) "a His-tagged recombinant protein fused to rabbit Fc tag", lot 3474103, dilution 1:5000; secondary anti-mouse HRP Bio-Rad, lot unknown, reference 1706516, dilution 1:5000; secondary anti-rabbit HRP Bio-Rad, lot unknown, reference 1706516, dilution 1:5000 |
| Validation | "Myc-Tag (9B11) Mouse mAb detects recombinant proteins containing the Myc epitope tag. The antibody recognizes the Myc-tag fused to either the amino or carboxy terminus of targeted proteins in transfected cells. The antibody may cross-react with c-myc protein. The antibody may weakly cross-react with a protein of unknown origin ~90kDa." https://www.cellsignal.de/products/primary-antibodies/myc-tag-9b11-mouse-mab/2276<br><br>Anti-RPS9 antibody was validated by the side-by-side western blot analysis of the WT yeast strain and a strain with tagged RPS9 protein (Raue et al., JBC 2007; Zhang et al., Nat Commun 2021)<br><br>"Anti-pan-ADP-ribose binding reagent is useful for the affinity detection of both mono- and poly-ADP-ribosylated proteins on membranes in a manner similar to antibody-based Western and dot blot analysis"  https://www.merckmillipore.com/DE/en/product/Anti-pan-ADP-ribose-binding-reagent,MM_NF-MABE1016" |

## Eukaryotic cell lines

Policy information about cell lines

| | |
|---|---|
| Cell line source(s) | BTI-Tnao38, species of origin - Trichoplusia ni. Provider - Boyce Thompson Institute for Plant Research, Inc., 533 Tower Road, Ithaca, NY 14853 |
| Authentication | The BTI-Tnao38 cell line was not authenticated. |
| Mycoplasma contamination | The cells were not tested for mycoplasma contamination. |
| Commonly misidentified lines (See ICLAC register) | BTI-Tnao38. The cell line is a commonly used system to produce recombinant proteins. |
